# Efficiency and safety of surgical intervention to patients with Non-Cystic Fibrosis bronchiectasis: a meta-analysis

**DOI:** 10.1038/srep17382

**Published:** 2015-12-02

**Authors:** Li-Chao Fan, Shuo Liang, Hai-Wen Lu, Ke Fei, Jin-Fu Xu

**Affiliations:** 1 Department of Respiratory Medicine, Shanghai pulmonary Hospital, Tongji University School of Medicine, Shanghai, China; 2 Department of Thoracic Surgery, Shanghai pulmonary Hospital, Tongji University School of Medicine, Shanghai, China

## Abstract

No quantitative systematic review was found to report the efficiency and safety of surgical resection in the management of non-cystic fibrosis (non-CF) bronchiectasis. We therefore conducted a meta-analysis to assess the effects of operative intervention to patients with non-CF bronchiectasis. PubMed, the Cochrane library and Web of Science databases were searched up to July 8th, 2015. The pooled mortality from 34 studies recruiting 4788 patients was 1.5% (95% CI, 0.9–2.5%). The pooled morbidity from 33 studies consisting of 4583 patients was 16.7% (95% CI, 14.8–18.6%). The pooled proportion of patients from 35 studies, consisting of 4614 patients who were free of symptoms was 66.5% (95% CI, 61.3–71.7%) after surgery. The summary proportion of patients from 35 articles including 4279 participants who were improved was 27.5% (95% CI, 22.5–32.5%), and 9.1% (95% CI, 7.3–11.5%) showed no clinical improvement. In conclusion, our analysis indicated that lung resection in the management of non-CF bronchiectasis is associated with significant improvements in symptoms, low risk of mortality and acceptable morbidity.

Non-cystic fibrosis (non-CF) bronchiectasis is a respiratory disease characterized by irreversible dilation of bronchi and persistent airway inflammation as a result of a vicious circle of impaired clearance of mucus and bacterial infection^1^. Patients with non-CF bronchiectasis suffer from chronic cough, purulent sputum, recurrent exacerbations, and progressive airway destruction^2^,^3^. The prevalence of non-CF bronchiectasis was 1,106 cases per 100,000 people from 2000 to 2007 in the United States, with an average annual percentage increase of 8.7%^4^. Compared with developed countries, the prevalence is even higher among developing countries^5^. There was a 2.5- to 3.9-fold higher period prevalence in Asians compared with whites and blacks^4^. A cross-sectional survey conducted in seven cities in China showed that the prevalence of non-CF bronchiectasis was 1.2 per 100 residents aged 40 years old or above^6^.

Therapies aimed at relieving cough, enhance mucous clearance, reducing exacerbations and improving quality of life. Besides medical treatment and respiratory physiotherapy, surgical treatment is an eligible therapy for patients with non-CF bronchiectasis, especially to those with little response to conservative interventions^7^. Over the years, many studies have reported surgical intervention in the management of patients with non-CF bronchiectasis^8^,^9^,^10^,^11^,^12^,^13^,^14^,^15^,^16^,^17^,^18^,^19^,^20^,^21^,^22^,^23^,^24^,^25^,^26^,^27^,^28^,^29^,^30^,^31^,^32^,^33^,^34^,^35^,^36^,^37^,^38^,^39^,^40^,^41^,^42^,^43^,^44^,^45^. The surgical procedure was aimed at primary resection of the affected area, as assessed by high-resolution computed tomographic (HRCT). Despite advances in thoracic surgery, surgical treatment of bronchiectasis remains controversial. Until now, no quantitative systematic review has investigated the efficiency and safety of surgical resection for the management of non-CF bronchiectasis, which prompted us to systematically assess the effects of operative intervention to patients with non-CF bronchiectasis. The present meta-analysis was undertaken to determine the efficiency and safety of surgical therapy for non-CF bronchiectasis patients. Efficiency was evaluated by symptom improvement after surgical treatment. Safety was evaluated by the incidence of post-operative mortality and morbidity. Morbidity was defined as post-operative complications, primarily including air leak more than seven days, atelectasis, hemorrhage, bronchopleural fistula, empyema, wound infection, cardiac arrhythmias and pneumonia. The primary outcomes include post-operative morbidity and mortality. The secondary outcomes were to evaluate symptom changes after lung resections to bronchiectasis at follow-up. The post-operative outcomes of symptom changes were categorized as “asymptomatic” if they had no symptoms post-operatively; “improved” if they had symptoms but milder than the pre-operative period and “no change or worsen”.

## Methods

### Search strategy

In order to identify all eligible studies, a comprehensive literature search of PubMed, Web of Science and Cochrane library was performed up to July 8th, 2015. The following terms were used: “surgery” or “operative” or “resection”, “bronchiectasis” or “NCFB” or “non-CF bronchiectasis”. The search was done on studies conducted on human subjects, without restriction on language. In addition, the references listed in each selected article were manually searched to identify additional eligible studies.

### Selection criteria

An eligible study should meet the following inclusion criteria: (1) It should be focused on surgical intervention in the management of patients with non-CF bronchiectasis. The diagnosis of bronchiectasis was based on HRCT scanning. Surgical procedures were focused on resection, regardless of incomplete or complete resections. Video assisted thoracoscopic surgery (VATS) and thoracotomy were both included. Type of surgical procedure included a lobectomy, followed by lobectomy and segmentectomy, pneumonectomy, a bilobectomy and segment resection. (2) It should provide the effect size of mortality or morbidity or symptomatic changes or complications. Operative mortality included patients who died within 30 days or those who died later but during the same hospitalization. Morbidity was defined as post-operative adverse events, such as residual pleural space, prolonged air leak, atelectasis, bronchopleural fistula, excessive bleeding, respiratory insufficiency and empyema. (3) The type of studies was not limited, regardless of prospective, retrospective or cross-sectional studies. Studies were excluded if one of the following existed: (1) Review articles and editorials; (2) Case reports; (3) Patients with chronic respiratory conditions other than non-CF bronchiectasis, such as cystic fibrosis, COPD and asthma; (4) Type of surgery was focused on lung transplantation. As lung transplant for recipients is indicated for rapidly deteriorating patients, these patients were more special, which is out of our consideration; (5) Data could not extract with current mathematical methods.

### Data extraction

Two investigators reviewed the eligible studies independently and any disagreement was settled by a third judge. The following information was extracted from each eligible paper: the first author’s name, year of publication, participants’ geographic location, average age of the population, symptom duration and follow-up period. Sample size as well as study design for each study were also summarized in supplementary material. After data extraction, discrepancies were adjudicated by discussion until a consensus was reached.

### Statistical analysis

Meta-analysis was performed with software STATA 12.0 and MetaAnalyst 3.13. The point estimates of effect size including the mortality and morbidity, rate of symptom changes and complications of operative intervention to non-CF bronchiectasis, and its 95% confidence interval (95% CI) were estimated for each study. We assessed the within and between study variation of heterogeneity by testing Cochran’s Q-statistic. The *Q* test and *I*^2^ statistics were used to assess the statistical heterogeneity between the studies^46^. If a difference in statistical heterogeneity was detected (*p* < 0.10 or *I*^2^ ≥ 50%), a random-effects model was applied. Otherwise, a fixed-effects model was used. The overall or pooled estimate of effect size was obtained using the Mantel and Haenszel method in the fixed effect model^47^ and using the DerSimonian and Laird method in the random effect model^48^. The calculation of the pooled effect size in the meta-analysis was performed weighting individual effect sizes by the inverse of their variance. If substantial heterogeneity was identified, subgroup analysis was performed to explore heterogeneity. Additionally, publication bias was examined by Egger’s test^49^. If the *p* value of the Egger’s test was <0.05 and the funnel plot was asymmetrical, publication bias was statistically significant. Otherwise, the study was considered to have no publication bias. If publication bias was suspected, we used Duval’s trim and fill method to correct for the bias^50^,^51^.

## Results

### Literature Search

**Figure 1:**
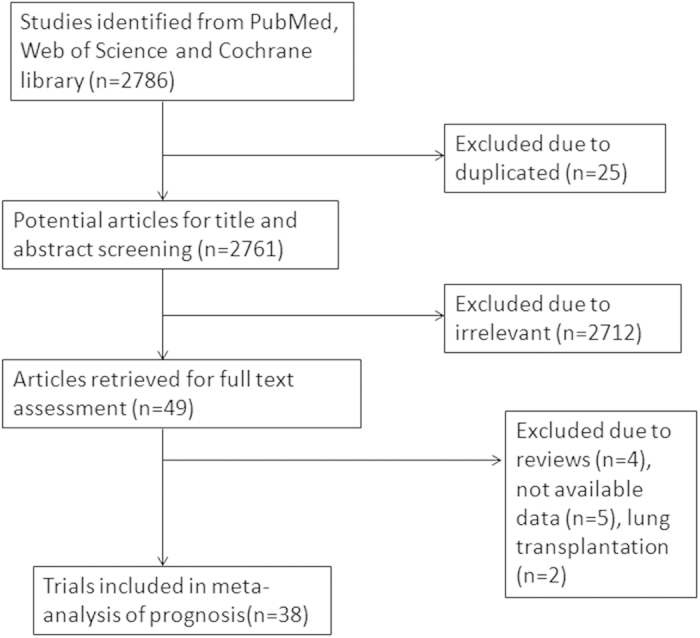
Flow chart of study selection.

Our initial search identified 2786 potentially relevant studies (2519 in PubMed, 258 studies in Web of Science and 9 articles in the Cochrane library), of which 25 studies were removed because of duplication. After screening titles and abstracts, 2712 were excluded due to lack of relevance. The remaining 49 papers were considered potentially eligible for full text review. Of these, 38 studies were included in the meta-analysis (Fig. 1).

### Characteristics of included studies

Characteristics of the eligible studies were summarized in Table 1 and Supplementary table S1. We identified a total of 5541 participants among 38 studies^8^,^9^,^10^,^11^,^12^,^13^,^14^,^15^,^16^,^17^,^18^,^19^,^20^,^21^,^22^,^23^,^24^,^25^,^26^,^27^,^28^,^29^,^30^,^31^,^32^,^33^,^34^,^35^,^36^,^37^,^38^,^39^,^40^,^41^,^42^,^43^,^44^,^45^ published from 1969 to 2014. Both children and adult patients were included. The range of enrollment periods for participants across studies was from 1937 to 2013. Patients were either infected with tuberculosis or other pathogens. Thirty studies provided the information on symptom duration. The mean symptom duration ranged from 1.45 to 14.9 years. The main symptoms at presentation were chronic productive cough, fetid sputum, repeated or massive hemoptysis, fever, empyema and chest pain. Among them, 11 studies including 1258 patients were from developed countries and 27 studies including 4283 subjects from developing countries. The countries where the studies had been carried out were as follows: Turkey (n = 16), America (n = 2), France (n = 2), Portugal (n = 2), Brazil (n = 2), China (n = 3), Iran (n = 2), Japan (n = 1), Germany (n = 2), Egypt (n = 1), Bulgaria (n = 1), India (n = 1), Saudi Arabia(n = 1), England (n = 1) and Suisse (n = 1). Thirty-three studies provided follow-up period. The mean follow-up duration ranged from 9 months to 15 years.

### Morbidity and mortality

**Figure 2:**
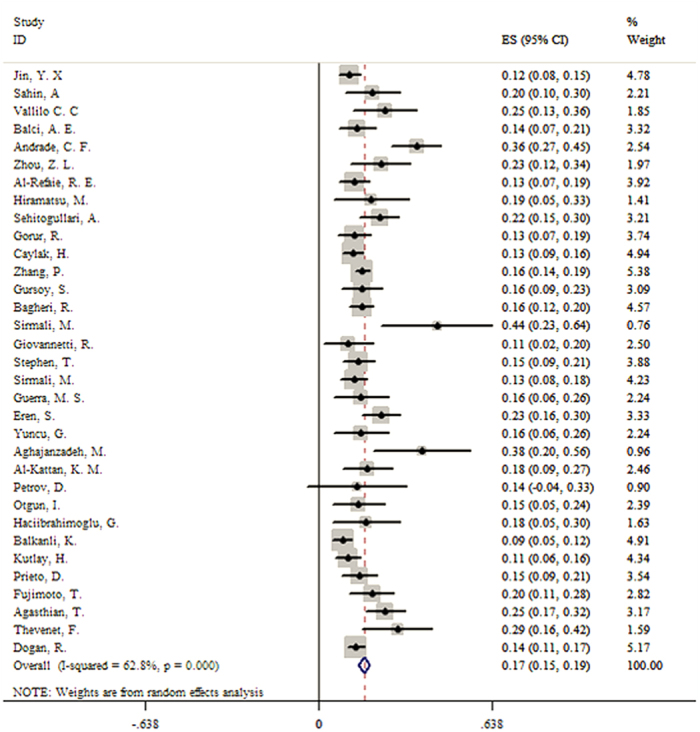
Forest plot assessed morbidity of surgery for the treatment of non-CF bronchiectasis.

**Figure 3:**
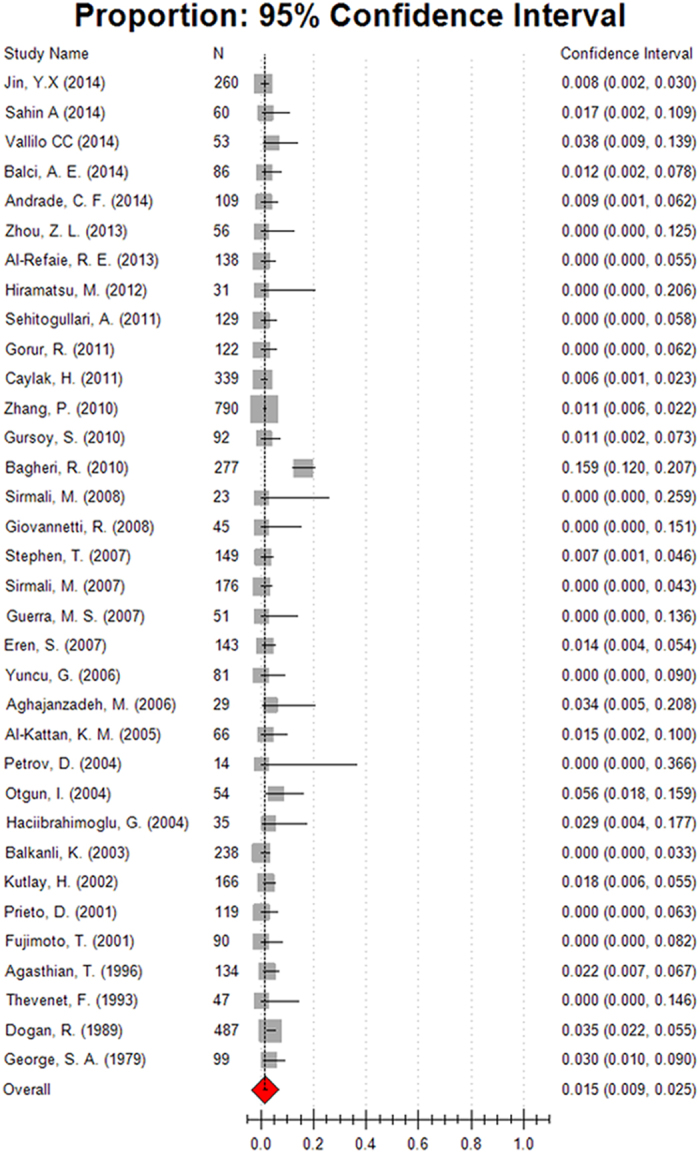
Forest plot assessed mortality of surgery for the treatment of non-CF bronchiectasis.

A total of 33 studies, consisting of 4583 patients reported morbidity of surgical intervention to non-CF bronchiectasis. The summary result was 16.7% (95% CI, 14.8–18.6%) (Fig. 2). Subgroup analyses showed that the morbidity was similar regardless of participants’ published year earlier or later than 2010, economy divided by developed countries and developing countries, geographic location and symptom duration. However, when stratified by age, the pooled morbidity was 16.2% (95% CI, 12.5–19.8%) for the 26 adults’ studies, relatively higher than the children’s studies (13.5%, 95% CI, 10.3–16.7%) (Table 2). We identified 34 articles recruited 4788 patients that permitted the pooling of results of mortality of surgical intervention to non-CF bronchiectasis. In our analyses, the pooled mortality was 1.5% (95% CI, 0.9–2.5%) (Fig. 3). Subgroup analyses showed that the summary results of children was 2.5% (95% CI, 1.2–5.3%) and 1.4% (95% CI, 0.8–2.5%) for adults. Compared with those who had longer symptom duration (more than five years), participants with short symptom duration had lower mortality (1.4%, 95% CI, 0.5–3.7%) (Table 2).

### Symptomatic changes

A total of 35 studies, consisting of 4614 patients, were included in the meta-analysis of proportion of asymptomatic after surgical intervention to bronchiectasis. Asymptomatic was defined as complete absence of pre-operative symptoms leading to surgery (symptom free patients). The summary result was 66.5% (95% CI, 61.3–71.7%). After seven potentially unpublished studies were filled, the pooled result shifted to 62.5% (95% CI, 57.4–67.7%) by using Duval’s trim and fill method. The pooled result of proportion of improvement after surgery was 27.5% (95% CI, 22.5–32.5%). Improvement was defined as reduction of preoperative symptoms, such as decrease volume of fetid sputum, recurrent hemoptysis and frequency of pulmonary infections (improved patients) (Table 3). However, there was still 9.1% (95% CI, 7.3–11.5%) proportion of patients who showed no improvement or worsen conditions (Supplemental table 2). The test for heterogeneity among studies was statistically significant (P < 0.05), so the random-effects model was used. To explore the possible causes of heterogeneity in the main analysis, we conducted additional subgroup analyses which were stratified by published year, economy, participants’ geographic location, age and symptom duration and follow-up period. Stratified by follow-up period, the proportion of asymptomatic was 73.3% (95% CI, 70.3–76.4%) for less than five years, relatively higher than more than five years (56.1%, 95% CI, 40.8–71.4%) (Table 3).

### Complications

The common post-operative complications were air leak, atelectasis, hemorrhage, bronchopleural fistula, empyema, wound infection, cardiac arrhythmias and pneumonia. Among 33 studies, the pooled outcome of any complication was 16.7% (95% CI, 14.8–18.6%). The proportion of air leak more than seven days and atelectasis after surgery was 3.6% (95% CI, 2.8–4.8%) and 3.61% (95% CI, 2.5–5.7%) respectively, higher than wound infection and cardiac arrhythmias (1.5%, 95% CI, 0.9–2.4% and 1.4%, 95% CI, 0.9–2.2%, respectively) (Table 4).

### Publication bias

Publication bias was assessed by Egger’s and Begg’s test. Funnel plot of 33 studies evaluating the morbidity of resection on bronchiectasis appeared to be symmetrical upon visual examination (Supplementary Figure 1). The data suggested that there was no evidence of publication bias (Begg’s test, p = 0.417, Eger’s test, p = 0.921). In the analysis of surgical mortality and symptom changes after surgery, the resultant funnel shape was asymmetrical, indicating publication bias (Supplementary Figure 2–5).

## Discussion

In this study, we sought to investigate the efficiency and safety of lung resection to patients with non-cystic fibrosis bronchiectasis by conducting meta-analyses. We included 38 articles covering 5541 subjects. Findings from meta-analyses showed that the operative morbidity and mortality were 16.7% and 1.5%, indicating that surgical intervention has an acceptable risk profile for patients with non-CF bronchiectasis. Follow-up results showed that 66.5% of patients became asymptomatic and 27.5% of patients alleviated symptoms after surgical treatment, suggesting surgery intervention for the treatment of non-CF bronchiectasis can serve as an effective treatment.

Studies reported surgical treatment for non-CF bronchiectasis was relatively rare in developed countries^15^,^23^,^26^,^37^,^38^,^39^,^40^,^41^,^42^,^43^,^44^,^45^, especially after the year of 2001, only three studies reported^15^,^23^,^26^. A possibility is that with the improvements in medical treatment and health insurance, the incidence of non-CF bronchiectasis is decreased in developed countries. However, despite the advent of antibiotics, it is still a significant clinical problem in developing countries because of tuberculosis, pneumonia, pertussis, and serious rubellainfections^14^,^52^.

The goal of therapy for non-CF bronchiectasis aims to relief symptoms and improve the quality of life^53^,^54^,^55^. Our study demonstrated that the effects of surgical intervention for treatment of non-CF bronchiectasis were notable, for most of the patients it diminished or eliminated symptoms after lung resection. The removal of diseased lung harboring persistent infection and inflammation would not only a benefit to adjacent healthy parenchyma of lung but also to patients’ systemic condition, which would prevent the progression of disease^7^. Vallilo and his colleages demonstrated that patients with symptomatic non-cystic fibrosis bronchiectais experience a significant improvement in their quality of life (QOL) after resection of compromised areas in the lung and exercise capacity after operation^8^. However, there was still 9.1% proportion of patients who showed no improvement or worse condition. It was reported that an immunocompromised status, *Pseudomonas aeruginosa* infection and the extent of residual bronchiectasis were independent and significant risk factors affecting the surgical outcome in the management of bronchiectasis^15^. Subgroup analyses showed that patients with symptom duration of more than five years had a relatively lower proportion of no improvement or worse condition, indicating that patients with a late stage of disease still could benefit from surgical treatment.

Subgroup analysis showed the pooled morbidity was a little higher among adults compared with children. A possible reason for surgical treatment in adults with higher morbidity maybe owing to more comorbidities in adults, such as diabetes and heart disease. Additionally, studies that reported operation treatment to children bronchiectasis were all after 2003. Over the years, the improvement of immunization and health care may also account for it. As for the mortality, it wasrelatively higher in children than in adults, possibly due to low immune maturity and tolerance to surgery compared to adults^12^. The mortality was relatively higher among patients whose symptom duration was more than five years, possibly, accounted forbacterial infection and extent of involved parenchyma. When stratified by published year, post-operative mortality was relatively lower in the recent five years. The improvement of health care and advance of medicine may account for it.

The safety issue is an important consideration when applying surgical intervention to non-CF bronchiectasis. The most addressed complication was air leak more than seven days and atelectasis, with a proportion of 3.6%. Failure to control peri-operative infection, causing accumulated secretion by intra-tracheal bacteria, might lead to atelectasis. Insufficient blood supply and electronic scalpel destroying the bronchial wall were risk factors of poor bronchial stump coverage, which were related with incidence of bronchopleural fistula^10^. Bronchopleural fistula could result in severe air leakage. Emergency operation and insufficient pre-operative preparation could also account for them. In contrast, cardiac arrhythmia was relatively less reported, the proportion was 1.4% (95% CI, 0.9–2.2%). Weaker physical condition of bronchiectasis patients and incomplete resection might be risk factors for post-operative infection. However, owing to the advent of antibiotics and the development of health care, the proportion of wound infection was only 1.5% (95% CI, 0.9–2.4%).

As a meta-analysis of published studies, our findings showed some advantages. First, we report here the first comprehensive meta-analyses of lung resection to non-CF bronchiectasis. Second, our study employed a large number of participants, allowing a much greater possibility of reaching conclusions between different surgical procedures.

There are some potential limitations in this meta-analysis. First of all, although we tried to collect all the relevant data, it is hard to ensure that no data was missed. However, we contacted the authors to require the missed information by email or telephone. Secondly, most of the studies were retrospective, only two were prospective studies. Despite the retrospective nature of the studies, we provided data on a large sample size of patients receiving surgery to treat bronchiectasis by meta-analyses and demonstrated the safety and efficacy of surgical intervention for treating this pathology. Thirdly, a significant heterogeneity was detected in the current meta-analyses. However, we used random effect model and did subgroup analyses. Duval and Tweedie’s trim-and-fill method were also carried out to adjust for publication bias. Fourthly, the enrolled patients of each study had different stages of disease and underwent different kinds of surgical intervention. It is hard to collect the outcome on certain type of patients with specific procedure of lung resection. It is uncertain whether procedure variabilitiesmay affect the outcome. Thus, larger number and higher quality of randomized controlled trials (RCTs) are still warranted.

## Conclusion

This meta-analysis provides further evidence that surgery for non-CF bronchiectasis is associated with a low risk of mortality and acceptable morbidity rates. Patients who fail to conservative treatment could consider surgical therapy.

## Additional Information

**How to cite this article**: Fan, L.-C. *et al*. Efficiency and safety of surgical intervention to patients with Non-Cystic Fibrosis bronchiectasis: a meta-analysis. *Sci. Rep*. **5**, 17382; doi: 10.1038/srep17382 (2015).

## Supplementary Material

Supplementary Information

## Figures and Tables

**Table 1:** Characteristics of the studies included in the meta-analysis.

Author	Year	Country	Age (years)	Symptom duration	Follow-up period
Jin, Y. X.	2014	China	30.2y	1 m–30y	6.7(3–10)y
Vallilo, C. C.	2014	Brazil	53(41.3 ± 12.9)y	NR	9 m
Sahin A	2014	Turkey	9.45 ± 4.12 (2–15)y	42.93 m	3.5 years
Balci, A. E.	2014	Turkey	37.8 ± 14.5 (11–73)y	43.4 ± 36.9 m	5.4 ± 3.2y(6 m–8.7 y)
Andrade, C. F.	2014	Brazil	7.6 y	NR	667 d
Zhou, Z. L.	2013	China	42.8(17–69) y	14.9(0.2–50)y	59.35(11–123 m)
Al-Refaie, R. E.	2013	Egypt	30.2 ± 15.7 y	9(3.7 ± 2.3)y	2.1 ± 1(1–4 y)
Hiramatsu, M.	2012	Japan	54 y	68(3–248)m	48(11–21 m)
Sehitogullari, A.	2011	Turkey	21.8 y	25.6 m	5.3y(1–8y)
Gorur, R.	2011	Turkey	22.3(14–44)y	7.2(1–18)y	2.8y(6 m-5)
Caylak, H.	2011	Turkey	22.4(15–50)y	39.3(1–216)m	13.6 m(3 m-10y)
Zhang, P.	2010	China	41.6(6–79 y)	36(2–360)m	4.2y(10 m-10y)
Gursoy, S.	2010	Turkey	38.7 ± 14.3 (10–67)y	5.2 ± 7.5y	15.3(1–60)m
Bagheri, R.	2010	Iran	34.7 y	2.5(1–14)y	4.5 years
Sirmali, M.	2008	Turkey	28(9–53)y	4.9(5 m-11)y	2.9(1.7–3.9)y
Giovannetti, R.	2008	France	42(9–68) y	22(1–72)m	52(2–96)m
Stephen, T.	2007	India	33.7(5–66) y	4.8y(3 m-12y)	4.8y(3 m-12y)
Sirmali, M.	2007	Turkey	12.3(3.4–16)y	3.8(0–7.6)y	4.3 y(14 m-7.2y)
Guerra, M. S.	2007	Portugal	38.6(4–65)y	3.4 y	3.4 y
Eren, S.	2007	Turkey	23.4(3–63)y	28.5(0–156)m	4.2 y(6 m-12y)
Yuncu, G.	2006	Turkey	24.4 y	NR	NR
Aghajanzadeh, M.	2006	Iran	30(5–60)y	NR	1 y(1–6y)
Karadag, B.	2005	Turkey	7.4 ± 3.7 y	4.9 ± 3.7(1–14.9) y	4.7 ± 2.7y(2–14 y)
Al-Kattan, K. M.	2005	Saudi Arabia	29.9 ± 10.8(6–55)y	3.4 ± 2.9(1–19)y	52 ± 9.4 m(24–82 m)
Petrov, D.	2004	Bulgaria	35.7 y	8.4 y	NR
Otgun, I.	2004	Turkey	9.25 ± 3.92(1.5–17)y	1.45 ± 3.81 y	48.37 ± 41.04(1–192 m)
Haciibrahimoglu, G.	2004	Turkey	10.6(5–13)y	4.2(1–9)y	5.4(1–12y)
Balkanli, K.	2003	Turkey	23.7(15–48)y	2.4(1–18)y	9 m(3 m-4y)
Kutlay, H.	2002	Turkey	34.1(7–70)y	5.7 y	4.2(1–10)y
Prieto, D.	2001	Portugal	42.2(11–77 y)	4(1–40)y	4.5y(1–10 y)
Fujimoto, T.	2001	Germany	44.7y(9–75)	10.6(0–50)y	6.1(0.6–10.8y)
Agasthian, T.	1996	America	48.4 (4–89 y)	6(1–60 y)	6 y(1–16 y)
Thevenet, F.	1993	France	36 ± 14 y	54 ± 58 m	54 ± 58 m
Etienne, T.	1993	Suisse	NR	NR	NR
Dogan, R.	1989	Turkey	25.5 y(2–56)	infancy/early childhood	4.6 y(4 m-10 y)
George, S. A.	1979	USA	44.5 y(6–63)	NR	10 y
Sanderson, J. M.	1974	England	NR	NR	1–15 y
Spath, F.	1969	Germany	6–67 y	NR	NR

Abbreviations: NR, not reported; y, years; m, months; d, day.

**Table 2:** Overall and subgroup meta-analyses of surgical morbidity and mortality to non-CF bronchiectasis.

subgroups	No. of studies	RR (95%CI)	Heterogeneity		Statistical method
			(I^2^%)	p	
Morbidity					
Published year <2010	19	0.156(0.127–0.184)	70.3	<0.001	random
Published year ≥2010	12	0.15(0.092–0.207)	95.6	<0.001	random
Developed countries	8	0.161(0.127–0.195)	69.8	0.002	random
Developing countries	23	0.154(0.117–0.192)	93.8	<0.001	random
Asian	23	0.156(0.118–0.194)	93.7	<0.001	random
European/Latin-America	8	0.161(0.127–0.195)	69.4	0.002	random
Children	5	0.135(0.103–0.167)	0	0.495	fixed
Adult	26	0.162(0.125–0.198)	93.6	<0.001	random
Symptom duration <5 y	15	0.153(0.102–0.205)	95	<0.001	random
Symptom duration ≥5 y	8	0.148(0.114–0.181)	65.9	<0.001	random
Overall	33	0.167(0.148–0.186)	92.8	<0.001	random
Mortality					
Published year <2010	20	0.024(0.018–0.033)	0	0.349	Fixed
Published year ≥2010	14	0.013(0.004–0.036)	47	<0.01	Random
Developed countries	8	0.018(0.009–0.034)	0	0.462	fixed
Developing countries	26	0.015(0.008–0.028)	45.3	<0.01	random
Asian	24	0.014(0.007–0.027)	45.6	<0.01	random
European/Latin-America	10	0.02(0.011–0.035)	0	0.457	fixed
Children	5	0.025(0.012–0.053)	22.1	0.188	fixed
Adult	29	0.014(0.008–0.025)	44.8	<0.01	random
Symptom duration <5 y	15	0.014(0.005–0.037)	46.7	<0.01	random
Symptom duration ≥5 y	10	0.023(0.016–0.034)	15.9	0.212	fixed
Overall	34	0.015(0.009–0.025)	44.1	<0.01	random

Abbreviations: 95% CI, 95% confidential interval; RR, risk ratio.

**Table 3:** Overall and subgroup meta-analyses of symptomatic improvement to non-CF bronchiectasis through surgery.

					Heterogeneity			
subgroups	No. of studies	RR (95%CI)	Z	P value	(I^2^%)	P	Egger p	Statistical method
Asymptomatic								
Published year <2010	23	0.628(0.547–0.708)	15.27	<0.01	94.9	<0.01	0.517	Random
Published year ≥2010	12	0.733(0.69–0.777)	32.92	<0.01	78.6	<0.01	0.965	Random
Developed countries	10	0.579(0.425–0.732)	7.39	<0.01	96.2	<0.01	0.148	Random
Developing countries	25	0.711(0.677–0.746)	40.64	<0.01	79.7	<0.01	0.115	Random
Asian/Africa	25	0.714(0.68–0.748)	41.37	<0.01	79.9	<0.01	0.151	Random
European/Latin–America	10	0.563(0.409–0.717)	7.16	<0.01	96	<0.01	0.277	Random
Children	5	0.585(0.440–0.730)	7.9	<0.01	86.2	<0.01	0.131	Random
Adult	26	0.715(0.68–0.749)	40.62	<0.01	78	<0.01	0.121	Random
Symptom duration <5 y	17	0.696(0.656–0.737)	33.65	<0.01	76.2	<0.01	0.458	Random
Symptom duration ≥5 y	11	0.689(0.624–0.754)	20.91	<0.01	86.4	<0.01	0.051	Random
Follow-up period <5 y	19	0.733(0.703–0.764)	47.61	<0.01	68.3	<0.01	0.872	Random
Follow-up period ≥5 y	12	0.561(0.408–0.714)	7.19	<0.01	96.7	<0.01	0.001	Random
overall	35	0.665(0.613–0.717)	25.18	<0.01	93.4	<0.01	0.007	Random
Alleviation								
Published year <2010	23	0.253(0.175–0.331)	6.35	<0.01	94.3	<0.01	0.067	Random
Published year ≥2010	12	0.238(0.167–0.310)	6.5	<0.01	94.7	<0.01	0.039	Random
Developed countries	10	0.385(0.253–0.517)	5.72	<0.01	95.2	<0.01	0.67	Random
Developing countries	25	0.227(0.183–0.271)	10.09	<0.01	90.4	<0.01	0.009	Random
Asian	23	0.193(0.164–0.222)	13.07	<0.01	76.1	<0.01	0.601	Random
European/Latin–America	12	0.415(0.284–0.547)	6.18	<0.01	95.6	<0.01	0.719	Random
Children	5	0.371(0.148–0.594)	3.27	0.001	96.1	<0.01	0.764	Random
Adults	26	0.202(0.173–0.231)	13.53	<0.01	76.7	<0.01	0.657	Random
Symptom duration <5y	20	0.213(0.182–0.244)	13.47	<0.01	69.7	<0.01	0.013	Random
Symptom duration ≥5 y	11	0.272(0.183–0.361)	5.99	<0.01	93.7	<0.01	0.01	Random
Follow-up period <5 y	21	0.227(0.179–0.275)	9.27	<0.01	90.3	<0.01	0.014	Random
Follow-up period ≥5 y	9	0.326(0.196–0.455)	4.92	<0.01	95	<0.01	0.013	Random
overall	35	0.275(0.225–0.325)	10.72	<0.01	94.2	<0.01	0.01	Random

Abbreviations: 95% CI, 95% confidential interval; RR, risk ratio.

**Table 4:** Complications after surgery for non-CF bronchiectasis.

Subgroups	No. of studies	Proportion(95% CI)	Tau-Squared	H	Q	Heterogeneity		Statistical method
						(I^2^%)	p	
Air leak >7 days	33	0.036(0.028–0.048)	0.229	0.592	0.985	34.4	0.001	Random
Atelectasis	33	0.036(0.025–0.057)	0.425	0.677	0.993	43.6	<0.01	Random
Hemorrhage	33	0.018(0.014–0.022)	0	0.468	0.961	0	0.45	Fixed
Bronchopleural fistula	33	0.016(0.011–0.022)	0.238	0.554	0.98	26.1	0.025	Fixed
Empyema	33	0.026(0.02–0.036)	0.25	0.585	0.985	33.2	0.001	Random
Wound infection	33	0.015(0.009–0.024)	0.467	0.622	0.989	38.7	<0.01	Random
Cardiac arrhythmias	33	0.014(0.009–0.022)	0.434	0.599	0.986	35.5	<0.01	Random
Pneumonia	33	0.023(0.018–0.030)	0.158	0.533	0.976	18.7	0.108	Fixed

Abbreviations: 95% CI, 95% confidential interval.
