# Supplementary material for: Efficiency and safety of surgical intervention to patients with Non-Cystic Fibrosis bronchiectasis: a meta-analysis
Source: Sci Rep. 2015 Dec 2;5:17382. doi: 10.1038/srep17382 (PMC4667173; doi:10.1038/srep17382)
Supplement: Supplementary Information [file srep17382-s1.pdf]

Efficiency and safety of surgical intervention to patients with Non-Cystic Fibrosis bronchiectasis: a meta-analysis

Authors: Li-Chao Fan [1], Shuo Liang [1], Hai-Wen Lu [1], Ke Fei[2$] & Jin-Fu Xu [1$]

Institutional affiliations:

1 Department of Respiratory Medicine, Shanghai pulmonary Hospital, Tongji University School of Medicine, Shanghai, China

2 Department of Thoracic Surgery, Shanghai pulmonary Hospital, Tongji University School of Medicine, Shanghai, China

$: Both authors contributed equally to this study.

Correspondence to: Jin-Fu Xu, MD, PhD, Department of Respiratory Medicine, Shanghai Pulmonary Hospital, Tongji University School of Medicine, No. 507 Zhengmin Road, Shanghai 200433, China; E-mail: jfxucn@gmail.com

Supplemental material:

Table S1 Characteristics of the studies included in the meta-analysis

Author	Year	Study design	Sample size (Total)	Male	Female
Jin, Y.X	2014	Retrospective	260	97	163
Sahin A	2014	Retrospective	60	32	28
Balci, A. E.	2014	Retrospective	86	58	28
Andrade, C. F.	2014	Retrospective	109	64	45
Vallilo, C.C	2014	Prospective	53	27	26
Zhou, Z. L.	2013	Prospective	56	26	30
Al-Refaie, R. E.	2013	Retrospective	138	77	61
Hiramatsu, M.	2012	Retrospective	31	2	29
Sehitogullari, A.	2011	Retrospective	129	84	45
Gorur, R.	2011	Retrospective	122	106	16
Caylak, H.	2011	Retrospective	339	301	38
Zhang, P.	2010	Retrospective	790	466	324
Gursoy, S.	2010	Retrospective	92	38	54
Bagheri, R.	2010	Retrospective	277	200	77
Sirmali, M.	2008	Retrospective	23	17	6
Giovannetti, R.	2008	Retrospective	45	21	24
Stephen, T.	2007	Retrospective	149	105	44
Sirmali, M.	2007	Retrospective	176	81	95
Guerra, M. S.	2007	Retrospective	51	29	22
Eren, S.	2007	Retrospective	143	91	52
Yuncu, G.	2006	Retrospective	81	47	34
Aghajanzadeh, M.	2006	Retrospective	29	22	7
Karadag, B.	2005	Retrospective	111	NA	NA
Al-Kattan, K. M.	2005	Prospective	66	34	32
Petrov, D.	2004	Retrospective	14	9	5
Otgun, I.	2004	Retrospective	54	24	20
Haciibrahimoglu, G.	2004	Retrospective	35	18	17
Balkanli, K.	2003	Retrospective	238	205	33
Kutlay, H.	2002	Retrospective	166	74	92
Prieto, D.	2001	Retrospective	119	48	71
Fujimoto, T.	2001	Retrospective	90	44	46
Agasthian, T.	1996	Retrospective	134	55	79
Thevenet, F.	1993	Retrospective	47	21	27
Etienne, T.	1993	Retrospective	85	NA	NA
Dogan, R.	1989	Retrospective	487	278	199
George, S. A.	1979	Retrospective	99	24	75
Sanderson, J. M.	1974	Retrospective	393	NA	NA
Spath, F.	1969	Retrospective	164	NA	NA

Table S2. Overall and Sub-group meta-analysis of no improvement or worse to surgical bronchiectasis.

Subgroups	No. of studies	Rate (95%CI)	Tau-Squared	H	Heterogeneity		Statistical method
					(I^2%)	p	
Sample size < 100	21	0.107(0.076-0.149)	0.325	0.654	41.9	<0.01	random
Sample size≥ 100	14	0.075(0.054-0.103)	0.246	0.715	45.7	<0.01	random
Published year≥2010	12	0.086(0.061-0.120)	0.222	0.691	44.4	<0.01	random
Published year< 2010	23	0.094(0.067-0.130)	0.36	0.69	44.4	<0.01	random
Developed countries	10	0.106(0.074-0.15)	0.198	0.635	40.1	0.001	random
Developing countries	25	0.086(0.064-0.115)	0.315	0.705	45.2	<0.01	random
Asian	23	0.082(0.06-0.112)	0.327	0.711	45.5	<0.01	random
European/Latin-America	12	0.112(0.082-0.152)	0.177	0.622	38.7	0.002	random
Children	6	0.117(0.067-0.196)	0.292	0.651	41.6	0.004	random
Adult	25	0.078(0.058-0.105)	0.308	0.7	44.9	<0.01	random
Symptom duration<5y	19	0.098(0.074-0.129)	0.299	0.701	45	<0.01	random
Symptom duration≥5y	9	0.079(0.051-0.119)	0.232	0.64	40.6	0.001	random
Follow up period<5y	18	0.075(0.05-0.109)	0.381	0.732	46.4	<0.01	random
Follow up period≥5y	13	0.133(0.114-0.155)	0.062	0.556	26.6	0.085	random
Overall	35	0.091(0.073-0.115)	0.277	0.687	44.2	<0.01	random

Abbreviations: 95%CI ,95% confidential interval.

Supplemental figure legends

Figure S1. Funnel plot of surgical morbidity for the treatment of non-CF bronchiectasis.

Figure S2. Funnel plot of surgical mortality for the treatment of non-CF bronchiectasis.

Figure S3. Funnel plot of asymptomatic to surgical bronchiectasis by using Duval’ s trim and fill method.

Figure S4. Funnel plot of improvement to surgical bronchiectasis.

Figure S5. Funnel plot of no improvement or worsen to surgical bronchiectasis.

Figure S1.

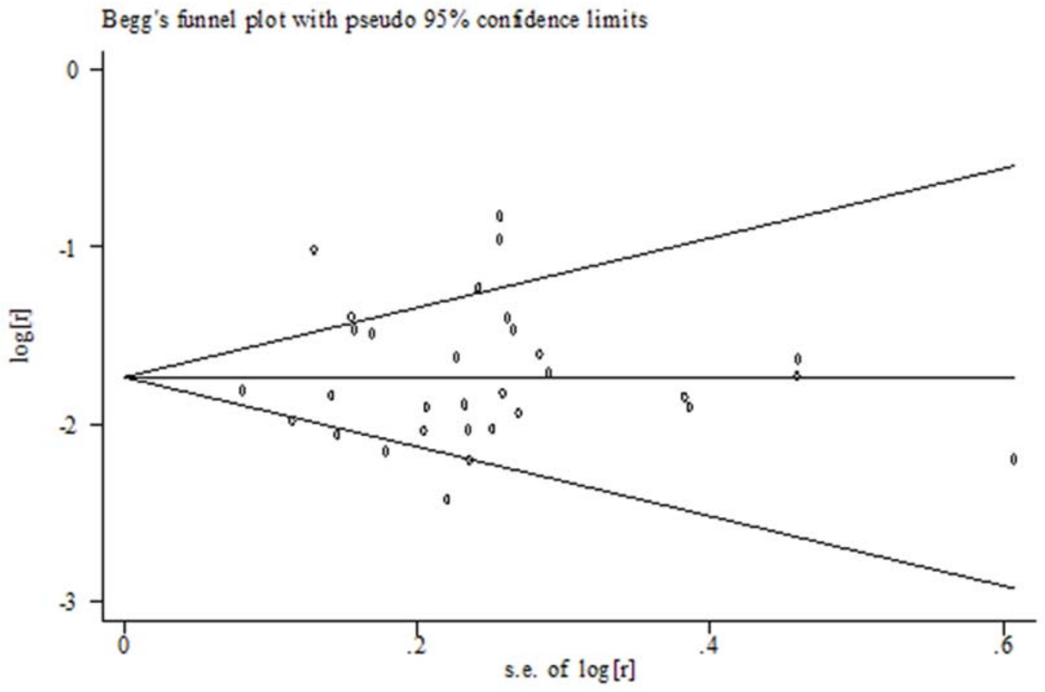

Figure S2.

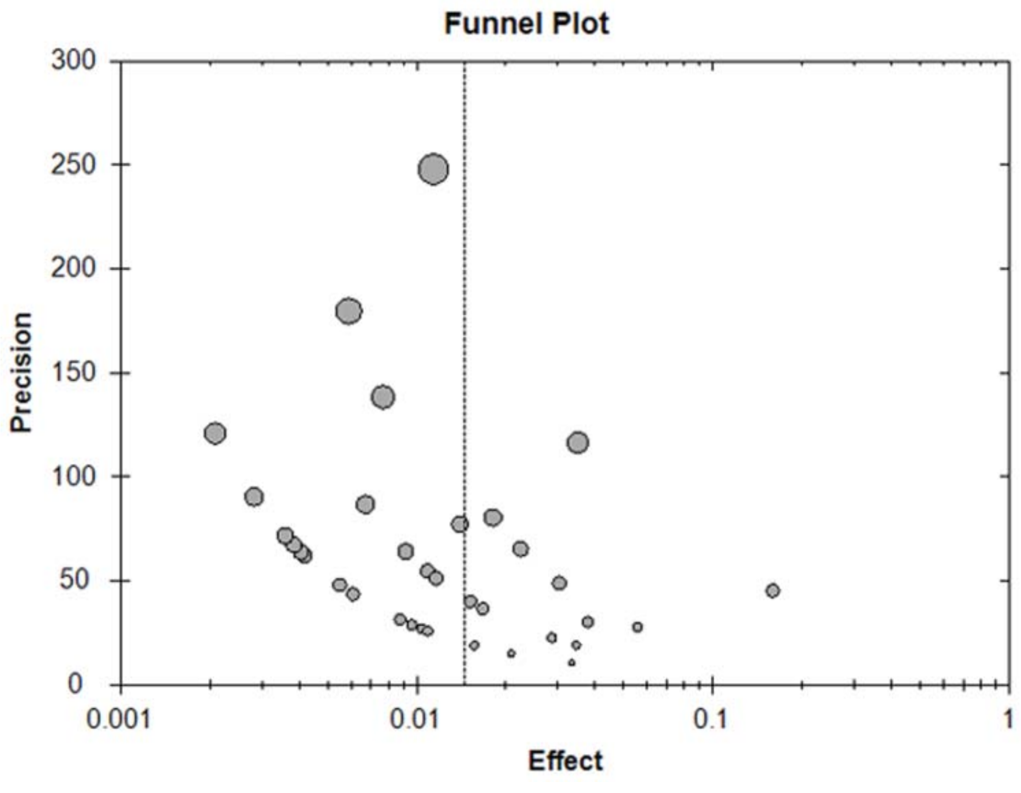

Figure S3.

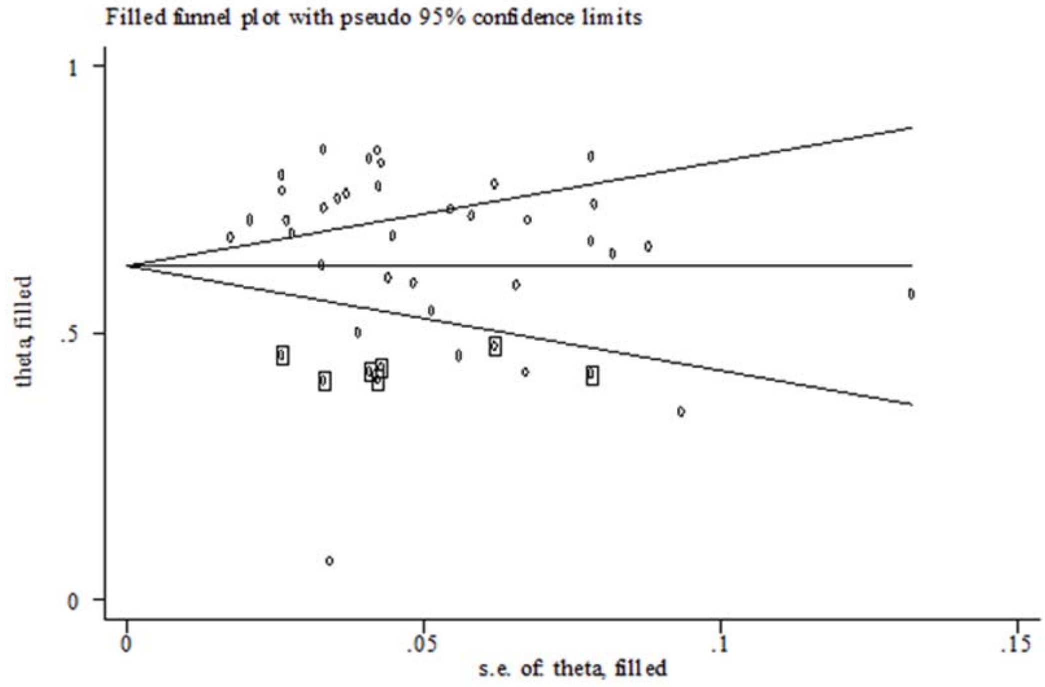

Figure S4.

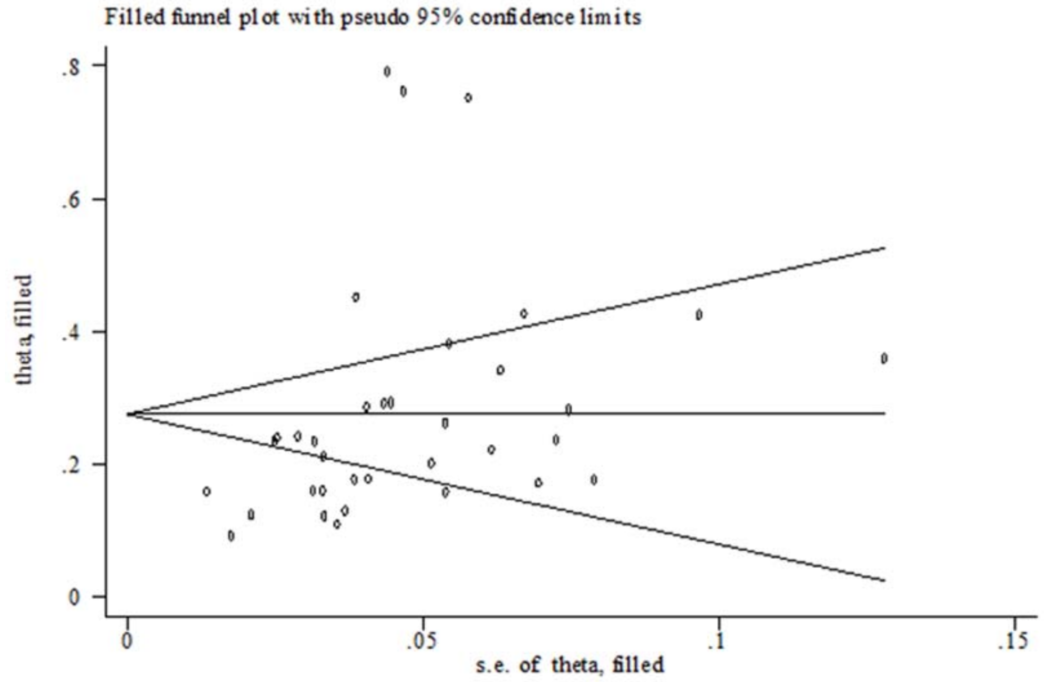

Figure S5.

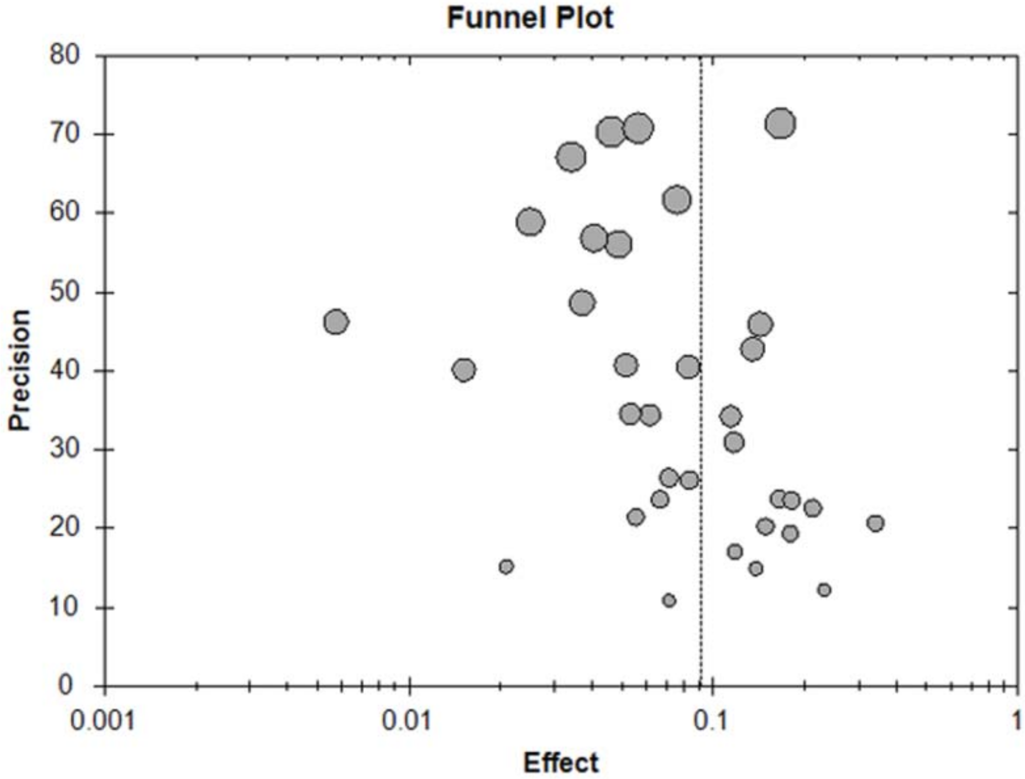
Funnel Plot
Precision
Effect
0
10
20
30
40
50
60
70
80
0.001
0.01
0.1
1
